# Ultra-processed food consumption and metabolic disease risk: an umbrella review of systematic reviews with meta-analyses of observational studies

**DOI:** 10.3389/fnut.2024.1306310

**Published:** 2024-01-31

**Authors:** Jia-Le Lv, Yi-Fan Wei, Jia-Nan Sun, Yu-Chen Shi, Fang-Hua Liu, Ming-Hui Sun, Qing Chang, Qi-Jun Wu, Yu-Hong Zhao

**Affiliations:** ^1^Department of Clinical Epidemiology, Shengjing Hospital of China Medical University, Shenyang, China; ^2^Clinical Research Center, Shengjing Hospital of China Medical University, Shenyang, China; ^3^Liaoning Key Laboratory of Precision Medical Research on Major Chronic Disease, Shengjing Hospital of China Medical University, Shenyang, China; ^4^Department of Obstetrics and Gynecology, Shengjing Hospital of China Medical University, Shenyang, China

**Keywords:** ultra-processed food, metabolic diseases, meta-analysis, umbrella review, observational study

## Abstract

**Background and aims:**

There is an ongoing debate on whether to advocate reducing ultra-processed food (UPF) in dietary guidelines to control metabolic disease (such as obesity and type 2 diabetes mellitus [T2DM]). We aimed to summarize the evidence from systematic reviews with meta-analyses between UPF consumption and metabolic diseases risk, assess the credibility, and verify the robustness of these associations.

**Methods:**

We systematically searched PubMed, Web of Science, Embase, and Cochrane Library databases from their inception to July 15, 2023, to identify relevant systematic reviews with meta-analyses. We used the random-effects model to evaluate the summary effect size, along with 95% confidence interval and prediction interval. We also assessed heterogeneity, evidence of small-study effects and excess significance bias, and categorized the credibility of each association based on quantitative umbrella review criteria. Additionally, we conducted subgroup and sensitivity analyses to assess the robustness of associations based on continents, study design, dietary assessment methods, definition methods of UPF, population, and units of UPF consumption.

**Results:**

Overall, 6 systematic reviews with 13 meta-analyses were included. Three (23.08%) meta-analyses were classified as highly suggestive evidence for meeting the criteria that associations were significant at *p* < 10^−6^, had more than 1,000 cases, and presented the largest study with significance at *p* < 0.05. Among them, the highest UPF consumption quantile was associated with an increased risk of obesity (OR = 1.55, 95% CI: 1.36–1.77) when compared with the lowest UPF consumption quantile. The highest UPF consumption quantile was associated with an increased risk of T2DM (RR = 1.40, 95% CI: 1.23–1.59) when compared with the lowest UPF consumption quantile, and a 10% increase in UPF consumption (% g/d) was associated with an increased risk of T2DM (RR = 1.12, 95% CI: 1.10–1.13). Meanwhile, the robustness of these associations was verified by a series of subgroup and sensitivity analyses.

**Conclusion:**

UPF consumption may be a risk factor for several metabolic diseases. However, well-designed studies are still needed to verify our findings in the future.

## Introduction

Metabolic disease is a metabolic disorder of organs, tissues, or cells caused by abnormal synthesis and decomposition of certain substances during metabolism, such as obesity and type 2 diabetes mellitus (T2DM) (1). It has become a serious burden on human society due to its rapidly increasing incidence worldwide (2–4). Currently, the precise etiology of metabolic disease is not fully understood, both genetic and environmental factors play crucial roles in the occurrence and development of disease (5, 6). Among them, as one of the most important modifiable environmental factors, the role of diet factors on metabolic disease has received extensive attention (7, 8).

Traditional methods to improve health focused on nutrients as the key determinants of a healthful diet (9). However, this classical nutrient-centric view has been challenged by the NOVA classification system, which is proposed as a novel way to classify foods based on the degree of processing rather than nutritional components (9, 10). According to the NOVA classification system, ultra-processed food (UPF) is a group of foods defined as industrial formulations created mostly or entirely from substances extracted from foods, with additives and with little if any intact food, such as fast foods, savory snacks, cakes, soft and/or sweetened drinks, and sausages (10). In developed countries, UPF has become an important source of energy intake, and the percentage of total energy from UPF could even be more than 50% (11, 12). Several systematic reviews with meta-analyses suggested that UPF consumption was associated with various metabolic disease, such as obesity, T2DM, hypertension, non-alcoholic fatty liver disease (NAFLD), and metabolic syndrome (MetS) (13–18). Therefore, there is a view that it is necessary to advocate the reduction of UPF in dietary guidelines to optimize health and policies (19). However, there is also an opposite view questioning the significance of UPF (20), suggesting that the concept and investigations of UPF are vague, and the association between UPF consumption and metabolic disease such as obesity remain uncertain due to the existence of potential biases, therefore, the mention of UPF in dietary guidelines can add little to existing nutrient profiling systems (21).

The contradictory views may cause confusion for clinicians and public health policymakers to make decisions. Therefore, we conducted an umbrella review (UR) across published systematic reviews with meta-analyses to evaluate the credibility as well as verify the robustness of associations between UPF consumption and metabolic disease.

## Methods

The UR is an approach used to provide an overview of published systematic reviews with meta-analyses on the same topic and evaluate the credibility of associations (22–24). In this UR, we strictly followed the Preferred Reporting Items for Systematic Reviews and Meta-analyses reporting guideline (Supplementary Table S1) and meta-analysis of Observational Studies in Epidemiology guidelines (Supplementary Table S2) (25, 26), and the protocol was registered in the International Prospective Register of Systematic Reviews (CRD42023427297).

### Search strategy

Two investigators (J-LL and Y-FW) systematically searched PubMed, Web of Science, Embase, and Cochrane Library databases from their inception to July 15, 2023, to identify systematic reviews with meta-analyses that evaluated the associations between UPF consumption and metabolic disease risk. The search strategy is shown in Supplementary Table S3. No language restrictions were used when selecting eligible articles. Furthermore, included studies were backward snowballed manually and forward snowballed using the Web of Science citation tracking feature to identify additional eligible studies (27, 28).

### Inclusion and exclusion criteria

Articles were included if they met the following PI[E]COS (Population, Intervention or Exposure, Comparison, Outcome, Study design) criteria: (1) Population: participants of different ages; (2) Intervention/Exposure: including UPF which was defined according to the NOVA classification system; (3) Comparison: highest/moderate vs. lowest, or dose–response analysis, etc.; (4) Outcome: metabolic disease risk (e.g., obesity, T2DM, or MetS); and (5) Study design: UR of systematic reviews with meta-analysis of observational studies (cohort, nested case–control, case–control, or cross-sectional studies, etc.).

Exclusion criteria: (1) genetic polymorphisms, laboratory, and animal studies; (2) systematic reviews without quantitative evaluations; (3) studies that could not obtain study-specific data, including effect sizes (odds ratio [OR], relative risk [RR], or hazard ratio [HR], etc.), 95% confidence interval (CI), and the number of cases or total population; and (4) studies that included less than three original studies. When more than one meta-analysis on the same association was eligible, only the latest meta-analysis was included (29). Of note, any comparison of exposure could be included and treated as a unique meta-analysis, such as highest/moderate vs. lowest, and dose–response analysis (30–32). Moreover, when a systematic review reported meta-analyses on more than one eligible outcome, they were all included and assessed separately (22). Two investigators (J-LL and Y-FW) independently screened the titles and abstracts of identified records and selected eligible articles by scrutinizing the full text. Any disagreement in the results comparison process was resolved through consensus with a third investigator (Q-JW).

### Data extraction

Two investigators (J-NS and Y-CS) independently conducted data extraction, and any disagreement was resolved by consensus with the third investigator (Q-JW). From each eligible meta-analysis, we recorded the first author, year of publication, journal name, outcomes, number of studies included, and comparison (highest/moderate vs. lowest, or dose–response analysis, etc.). Regarding comparison, “lowest” was defined as the lowest UPF consumption quantile, “moderate” was defined as the first exposure quantile, and “highest” was defined as the highest UPF consumption quantile. For each original study, we extracted the first author, year of publication, country, study design (cohort, nested case–control, case–control, or cross-sectional studies, etc.), follow-up year, Newcastle-Ottawa Scale (NOS) score, diagnostic criteria for disease, dietary assessment methods (food frequency questionnaire [FFQ], 24-h dietary recall, or food record, etc.), definition methods (NOVA or non-NOVA) and units (% kcal/d, % g/d, or g/d, etc.) of UPF consumption, number of cases and participants, risk estimate (OR, RR, or HR) and 95% CI from the multivariable model, and covariates used for adjustment.

### Statistical analyses

For each association, we calculated the summary effect size and 95% CI using random effects methods (33). The 95% prediction interval (PI) was also calculated to account for between-study heterogeneity and to evaluate the possible range of the effect size in a new study addressing the same association (34). Between-study heterogeneity was evaluated by tau^2^ and *I*^2^ statistic (35). An *I*^2^ value <50, 50% ≤ to ≤75%, and > 75% were considered to represent not large, large, and very large heterogeneity, respectively (35). Egger’s regression asymmetry test was used to identify whether there was evidence for small-study effects (SSE) (i.e., whether smaller studies tend to report larger effect size than larger studies) (36). A *p*-value <0.10 with more conservative effects in the largest study (i.e., the study with the smallest standard error) than in random effects meta-analysis was considered to be evidence of SSE (37). We applied the excess statistical significance test to assess whether the number of observed studies (*O*) with statistically significant results was larger than the expected number of positive studies (*E*) (38). In each meta-analysis, we calculated *E* by the sum of the statistical power estimates for each component study (38). We evaluated the power of each component study using the effect size of the largest study, while a non-central *t* distribution was used to calculate the statistical power of each study (39). Excess significance bias (ESB) for each meta-analysis was denoted at *p* < 0.10 and *O* > *E* (38). Cohen’s kappa statistic was employed to evaluate the consistency of different procedures between the two investigators. The degree of consistency was explained by the kappa value (40).

In addition, subgroup analyses were performed to evaluate the robustness of results according to continents (America, Asia, or Europe), study design (prospective cohort, case–control, or cross-sectional studies), dietary assessment methods (FFQ, 24-h dietary recall, or food record), and definition methods (NOVA or non-NOVA) of UPF. Furthermore, sensitivity analyses were conducted in our UR. First, only one meta-analysis involved adolescent participants (15), therefore we excluded adolescent participants due to their limited representation in the included meta-analyses which mostly focused on adults. Second, in the meta-analyses that defined UPF entirely based on the NOVA classification system, we further considered the potential impact of UPF units and reanalyzed associations by excluding original studies that differed from the UPF units used in most original studies. All analyses were performed using STATA version 16 (StataCrop, College Station, TX, United States) and IBM SPSS Statistics version 21 (IBM Corp, Armonk, NY, United States).

### Grading the evidence

We applied quantitative criteria to categorize the credibility of each association into convincing, highly suggestive, suggestive, or weak according to previously published UR (Table 1) (23, 41, 42).

**Table 1:** The criteria to categorize the credibility of evidence in umbrella review.

Evidence class	Criteria
Convincing (Class I)	*p* < 10^−6^ under the random-effects model Number of cases >1,000 *p* < 0.05 of the largest study in the meta-analysis *I*^2^ < 50% 95% prediction interval that excluded the null value No evidence of small-study effects No evidence of excess significance bias
Highly suggestive (Class II)	*p* < 10^−6^ under the random-effects model Number of cases >1,000 *p* < 0.05 of the largest study in the meta-analysis
Suggestive (Class III)	*p* < 10^−3^ under the random-effects model Number of cases >1,000
Weak (Class IV)	*p* < 0.05 under the random-effects model
Not significant	*p* ≥ 0.05 under the random-effects model

### Quality assessment of evidence and methods

Two investigators (J-LL and M-HS) independently employed the Grading of Recommendations, Assessment, Development and Evaluation (GRADE) to assess the quality of each association (43). According to GRADE, the quality of each association was classified as high, moderate, low, or very low (43). For observational studies, the quality of evidence was initially classified as low, and then could be downgraded based on five factors including study limitations, imprecision, inconsistency, indirectness, and publication bias, and upgraded based on three factors including a large magnitude of effect, a dose–response gradient, and attenuation by plausible confounding (43).

In addition, two reviewers (J-LL and M-HS) independently used the Assessment of Multiple Systematic Reviews (AMSTAR) tool to assess the methodological quality of each included systematic review (44). AMSTAR evaluates 11 questions, with a maximum score of 11. Each meta-analysis was categorized as high, moderate, or low quality for scores of ≥8 points, 4–7 points, and ≤ 3 points, respectively (45).

## Results

### Literature review

**Figure 1:**
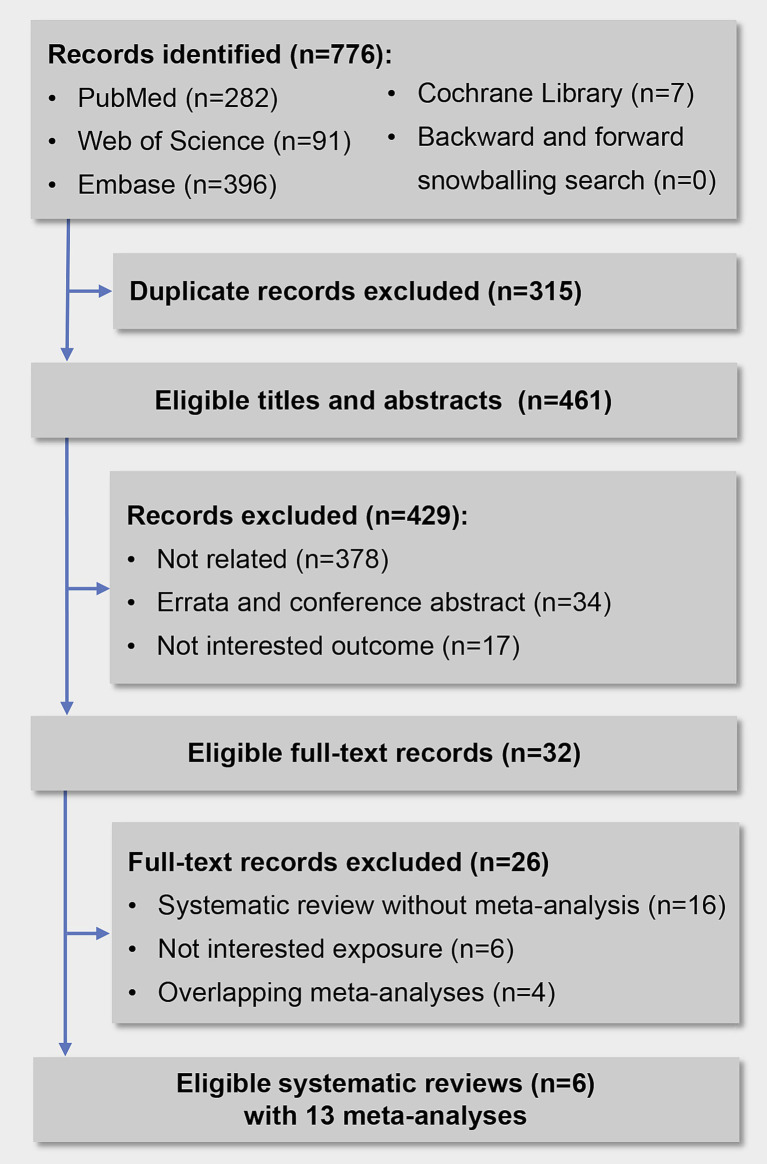
Flow diagram of the study selection process.

Overall, we initially identified 776 records, 315 records were excluded for duplication, and 429 records were excluded after screening titles and abstracts. Finally, we reviewed 32 full-text articles, and 6 systematic reviews with 13 meta-analyses met the inclusion criteria (Figure 1). Details of the 26 excluded articles are shown in Supplementary Table S4. The two investigators showed a high consistency in terms of data screening and selection, with a kappa value of 0.86.

### Characteristics of the included meta-analyses

The characteristics of 13 meta-analyses corresponding to 97 original studies are presented in Supplementary Table S5. Of the 13 meta-analyses, the median number of original studies was 7 (range from 3 to 18), that of participants was 66,235 (range from 15,152 to 992,242), and that of cases was 15,000 (range from 4,302 to 34,924). Regarding exposure, all meta-analyses involved UPF defined by the NOVA classification system, of which 3 (23.08%) meta-analyses also involved specific types of foods belonging to UPF. Data on UPF consumption were mainly collected using FFQ, 24-h dietary recall, and 4-day food records. In addition, the commonly used units of UPF consumption included % kcal/d and % g/d. Regarding outcome, we investigated a total of 6 outcomes, including abdominal obesity (13), hypertension (14), MetS (15), NAFLD (16), obesity (13), overweight (13), overweight and obesity (13), and T2DM (17, 18).

### UPF consumption and overweight and/or obesity risk

**Figure 2:**
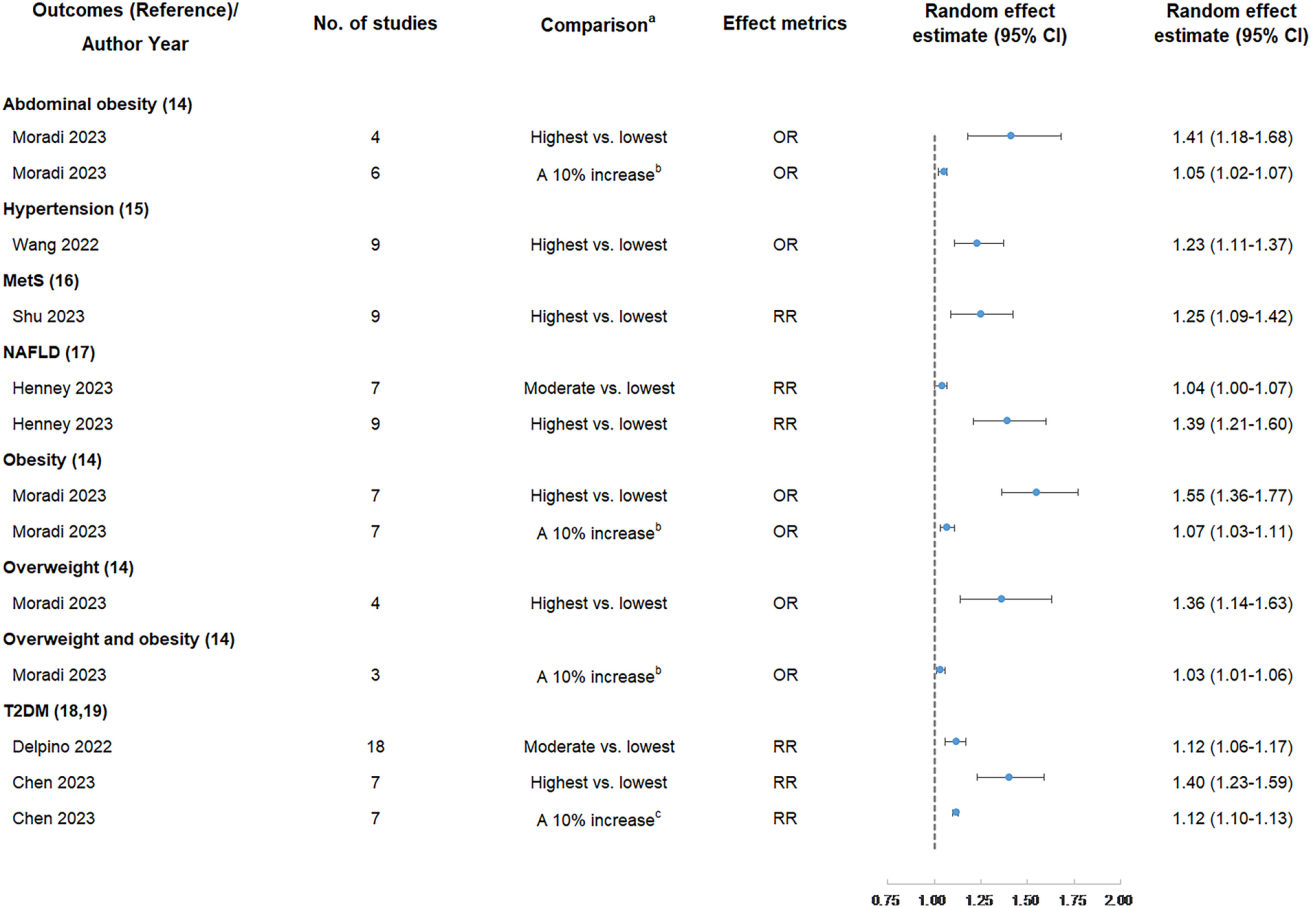
Summary random effect estimate with 95% confidence interval from 13 meta-analyses evaluating the association between ultra-processed food consumption and metabolic disease risk. CI, confidence interval, MetS, metabolic syndrome; NAFLD, non-alcoholic fatty liver disease; OR, odds ratio; RR, risk ratio; T2DM, type 2 diabetes mellitus. ^a^ “lowest” was defined as the lowest UPF consumption quantile, “moderate” was defined as the first exposure quantile, and “highest” was defined as the highest UPF consumption quantile. ^b^ The unit of UPF consumption was % kcal/d. ^c^ The unit of UPF consumption was % g/d.

**Figure 3:**
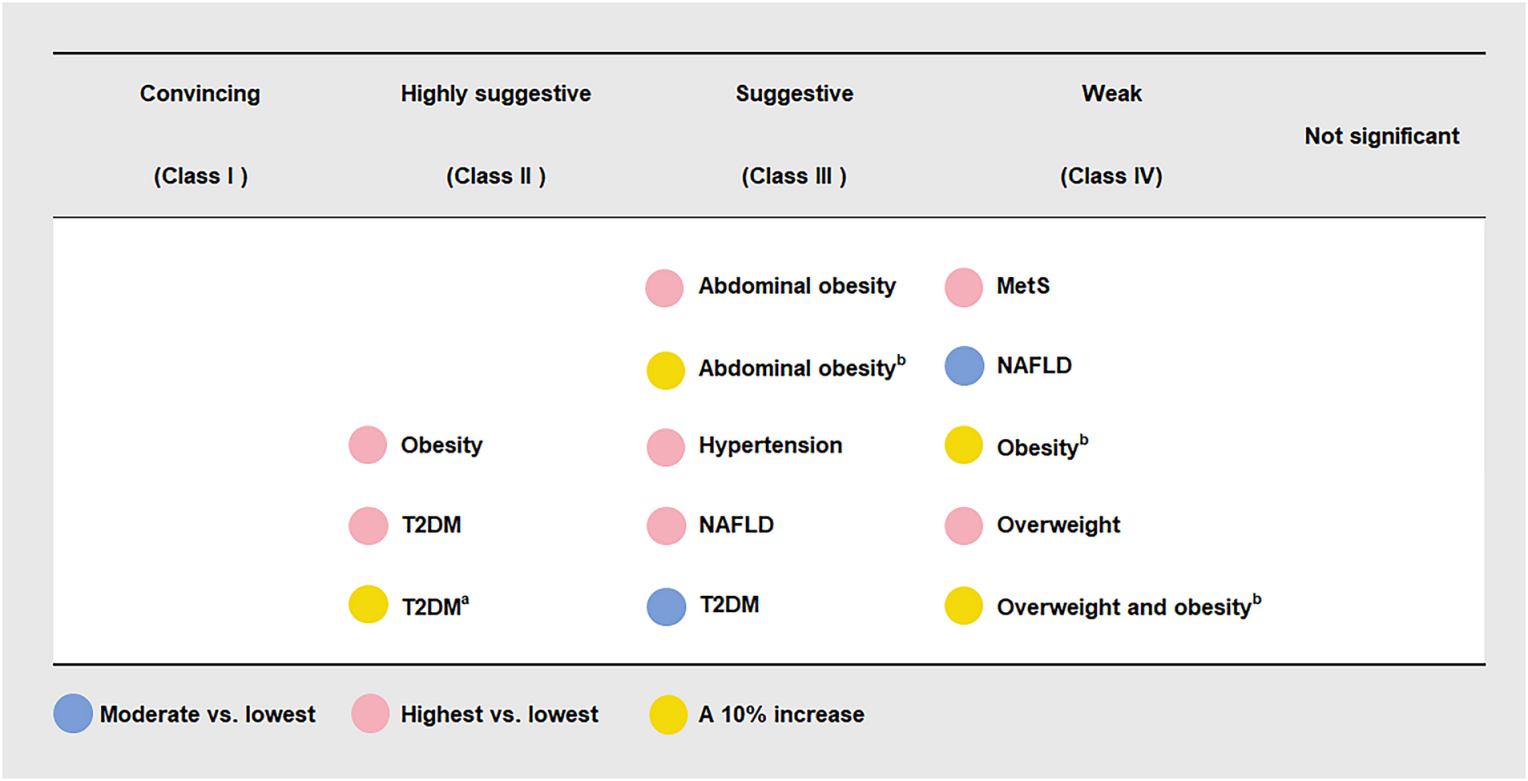
Credible assessment of 13 meta-analyses evaluating the association between ultra-processed food consumption and metabolic disease risk. MetS, metabolic syndrome; NAFLD, non-alcoholic fatty liver disease; T2DM, type 2 diabetes mellitus. “Lowest” was defined as the lowest UPF consumption quantile, “moderate” was defined as the first exposure quantile, and “highest” was defined as the highest UPF consumption quantile. ^a^ The unit of UPF consumption was % g/d. ^b^ The unit of UPF consumption was % kcal/d.

Six (46.15%) meta-analyses that evaluated the association between UPF consumption and overweight and/or obesity risk were all significant at *p* < 0.05. Among them, one meta-analysis was still significant at *p* < 10^−6^, had more than 1,000 cases, presented the largest study with significance at *p* < 0.05, and reported large heterogeneity, 95% PI excluding the null value, and evidence of ESB. According to the quantitative UR criteria, the above meta-analysis was classified as highly suggestive evidence, indicating that the highest UPF consumption quantile was associated with an increased risk of obesity (OR = 1.55, 95% CI: 1.36–1.77) when compared with the lowest UPF consumption quantile. In addition, according to criteria that the associations were still significant at *p* < 10^−3^ and had more than 1,000 cases, two meta-analyses were classified as suggestive evidence, demonstrating that the highest UPF consumption quantile was associated with an increased risk of abdominal obesity (OR = 1.41, 95% CI: 1.18–1.68) when compared with the lowest UPF consumption quantile, and a 10% increase in UPF consumption (% kcal/d) was associated with an increased risk of abdominal obesity (OR = 1.05, 95% CI: 1.02–1.07). The other three meta-analyses were classified as weak evidence, showing that the highest UPF consumption quantile was associated with an increased risk of overweight (OR = 1.36, 95% CI: 1.14–1.63) when compared with the lowest UPF consumption quantile, and a 10% increase in UPF consumption (% kcal/d) was associated with an increased risk of overweight and obesity (OR = 1.03, 95% CI: 1.01–1.06) as well as obesity (OR = 1.07, 95% CI: 1.03–1.11) (Figures 2, 3 and Table 2).

**Table 2:** Credible assessment of 13 meta-analyses evaluating the association between ultra-processed food consumption and metabolic disease risk.

Outcomes (Reference)/Author Year	No. of studies	Comparison^a^	Random *p*-value	No. of cases	95% PI	*I*^2^ (95% CI) (%)	Tau^2^	Largest study effect (95% CI)	SSE/ESB	Evidence class
**Abdominal obesity** (13)										
Moradi 2023	4	Highest vs. lowest	2.00 × 10^−4^	13,928	0.69–2.87	62 (0–87)	0.02	1.21 (1.10–1.46)	No/Yes	Suggestive
Moradi 2023	6	A 10% increase^b^	5.36 × 10^−5^	17,011	0.98–1.12	77 (47–89)	0.00	1.02 (1.01–1.03)	No/No	Suggestive
**Hypertension** (14)										
Wang 2022	9	Highest vs. lowest	1.37 × 10^−4^	13,375	0.92–1.64	52 (0–77)	0.01	1.21 (1.06–1.37)	No/Yes	Suggestive
**MetS** (15)										
Shu 2023	9	Highest vs. lowest	1.00 × 10^−3^	8,649	0.84–1.85	85 (73–92)	0.02	1.00 (0.99–1.01)	Yes/No	Weak
**NAFLD** (16)										
Henney 2023	7	Moderate vs. lowest	0.03	12,367	0.99–1.08	0 (0–71)	0.00	1.03 (0.99–1.07)	No/No	Weak
Henney 2023	9	Highest vs. lowest	2.65 × 10^−6^	12,977	0.90–2.17	89 (82–94)	0.03	1.05 (1.02–1.09)	Yes/No	Suggestive
**Obesity** (13)										
Moradi 2023	7	Highest vs. lowest	1.17 × 10^−10^	21,149	1.06–2.26	55 (0–81)	0.02	1.53 (1.29–1.81)	No/Yes	Highly suggestive
Moradi 2023	7	A 10% increase^b^	0.001	15,000	0.95–1.21	88 (79–94)	0.00	1.00 (0.99–1.01)	Yes/No	Weak
**Overweight** (13)										
Moradi 2023	4	Highest vs. lowest	1.00 × 10^−3^	16,131	0.65–2.87	73 (22–90)	0.02	1.13 (1.08–1.41)	No/No	Weak
**Overweight and obesity** (13)										
Moradi 2023	3	A 10% increase^b^	5.00 × 10^−3^	4,302	0.84–1.27	39 (0–81)	0.00	1.02 (1.00–1.04)	No/Yes	Weak
**T2DM** (17, 18)										
Delpino 2022	18	Moderate vs. lowest	7.63 × 10^−6^	34,924	1.00–1.25	24 (0–57)	0.00	1.21 (1.12–1.31)	No/No	Suggestive
Chen 2023	7	Highest vs. lowest	3.15 × 10^−7^	21,932	0.91–2.13	88 (78–94)	0.00	1.12 (1.04–1.20)	No/No	Highly suggestive
Chen 2023	7	A 10% increase^c^	5.44 × 10^−79^	21,932	1.10–1.14	2 (0–71)	0.02	1.13 (1.11–1.15)	No/Yes	Highly suggestive

CI, confidence interval; ESB, excess significance bias; MetS, metabolic syndrome; NAFLD, non-alcoholic fatty liver disease; PI, prediction interval; SSE, small-study effects; T2DM, type 2 diabetes mellitus.

a “Lowest” was defined as the lowest UPF consumption quantile, “moderate” was defined as the first exposure quantile, and “highest” was defined as the highest UPF consumption quantile.

b The unit of UPF consumption was % kcal/d.

c The unit of UPF consumption was % g/d.

### UPF consumption and T2DM risk

Three (23.08%) meta-analyses that evaluated the association between UPF consumption and T2DM risk were all significant at *p* < 10^−3^. Among them, two meta-analyses were still significant at *p* < 10^−6^, had more than 1,000 cases, and presented the largest study with significance at *p* < 0.05. According to the quantitative umbrella review criteria, they were classified as highly suggestive evidence, indicating that the highest UPF consumption quantile was associated with an increased risk of T2DM (RR = 1.40, 95% CI: 1.23–1.59) when compared with the lowest UPF consumption quantile, and a 10% increase in UPF consumption (% g/d) was associated with an increased risk of T2DM (RR = 1.12, 95% CI: 1.10–1.13). In addition, one meta-analysis was classified as suggestive evidence due to meeting the criteria of cases more than 1,000 simultaneously, demonstrating that moderate UPF consumption quantile was associated with an increased risk of T2DM (RR = 1.12, 95% CI: 1.06–1.17) when compared with the lowest UPF consumption quantile (Figures 2, 3 and Table 2).

### UPF consumption and NAFLD risk

Two (15.38%) meta-analyses that evaluated the association between UPF consumption and T2DM risk were all significant at *p* < 0.05. One meta-analysis was still significant at *p* < 10^−3^ and had more than 1,000 cases, classifying as suggestive evidence and demonstrating that the highest UPF consumption quantile was associated with an increased risk of NAFLD (RR = 1.39, 95% CI: 1.21–1.60) when compared with the lowest UPF consumption quantile. The other meta-analysis was classified as weak evidence, suggesting that moderate UPF consumption quantile was associated with an increased risk of NAFLD (RR = 1.04, 95% CI: 1.00–1.07) when compared with the lowest UPF consumption quantile (Figures 2, 3 and Table 2).

### UPF consumption and hypertension risk

One (7.69%) meta-analysis was classified as suggestive evidence according to *p* < 10^−3^ and cases more than 1,000, demonstrating that the highest UPF consumption quantile was associated with an increased risk of hypertension (OR = 1.23, 95% CI: 1.11–1.37) when compared with the lowest UPF consumption quantile (Figures 2, 3 and Table 2).

### UPF consumption and MetS risk

One (7.69%) meta-analysis was classified as weak evidence according to *p* < 0.05, suggesting that the highest UPF consumption quantile was associated with an increased risk of MetS (RR = 1.25, 95% CI: 1.09–1.42) when compared with the lowest UPF consumption quantile (Figures 2, 3 and Table 2).

### Findings of subgroup and sensitivity analyses

**Figure 4:**
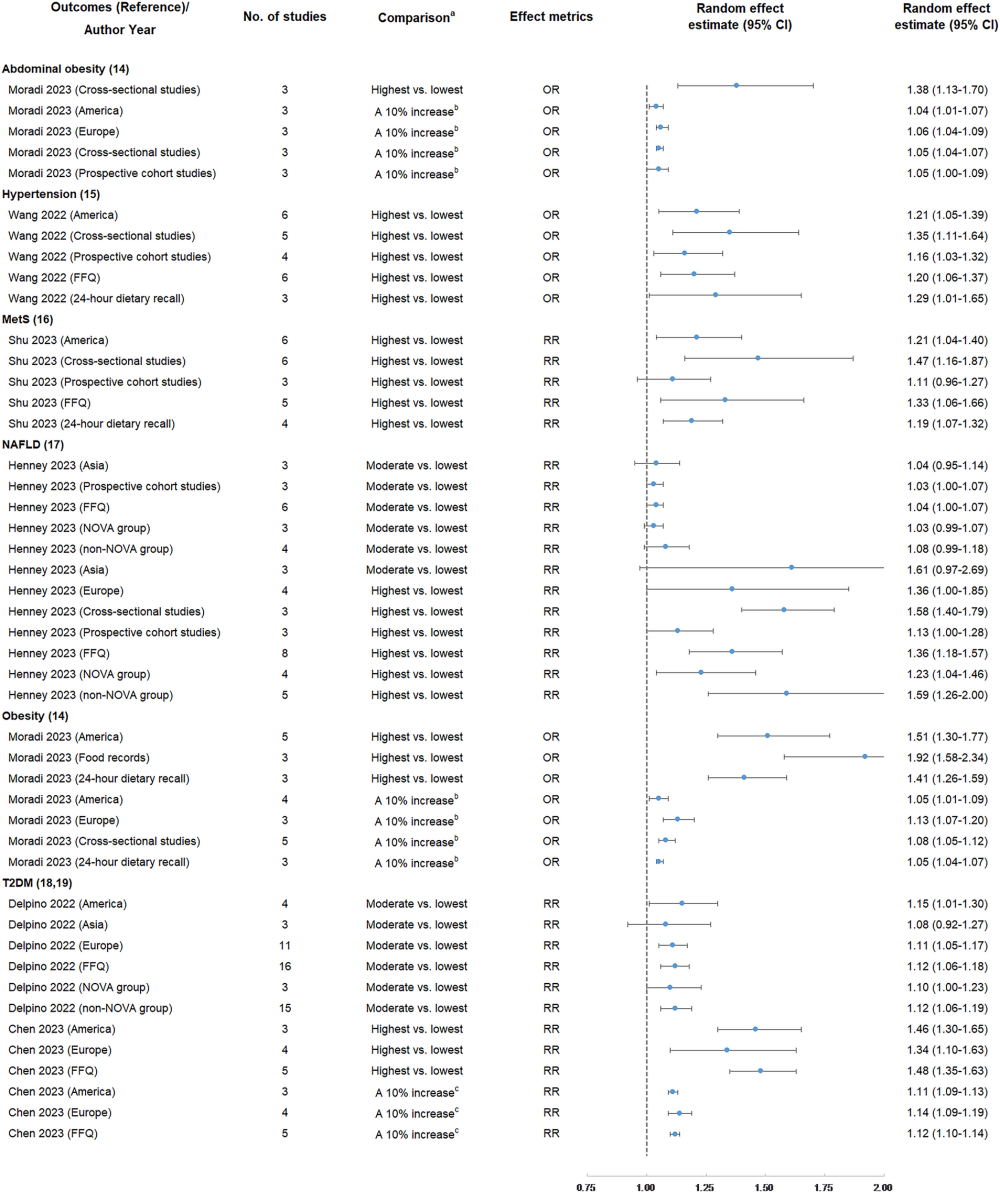
Subgroup analyses of summary random effect estimate with 95% confidence interval from meta-analyses evaluating the association between ultra-processed food consumption and metabolic disease risk. CI, confidence interval; FFQ, food frequency questionnaire; MetS, metabolic syndrome; NAFLD, non-alcoholic fatty liver disease; OR, odds ratio; RR, risk ratio; T2DM, type 2 diabetes mellitus. ^a^ “lowest” was defined as the lowest UPF consumption quantile, “moderate” was defined as the first exposure quantile, and “highest” was defined as the highest UPF consumption quantile. ^b^ The unit of UPF consumption was % kcal/d. ^c^ The unit of UPF consumption was % g/d.

**Figure 5:**
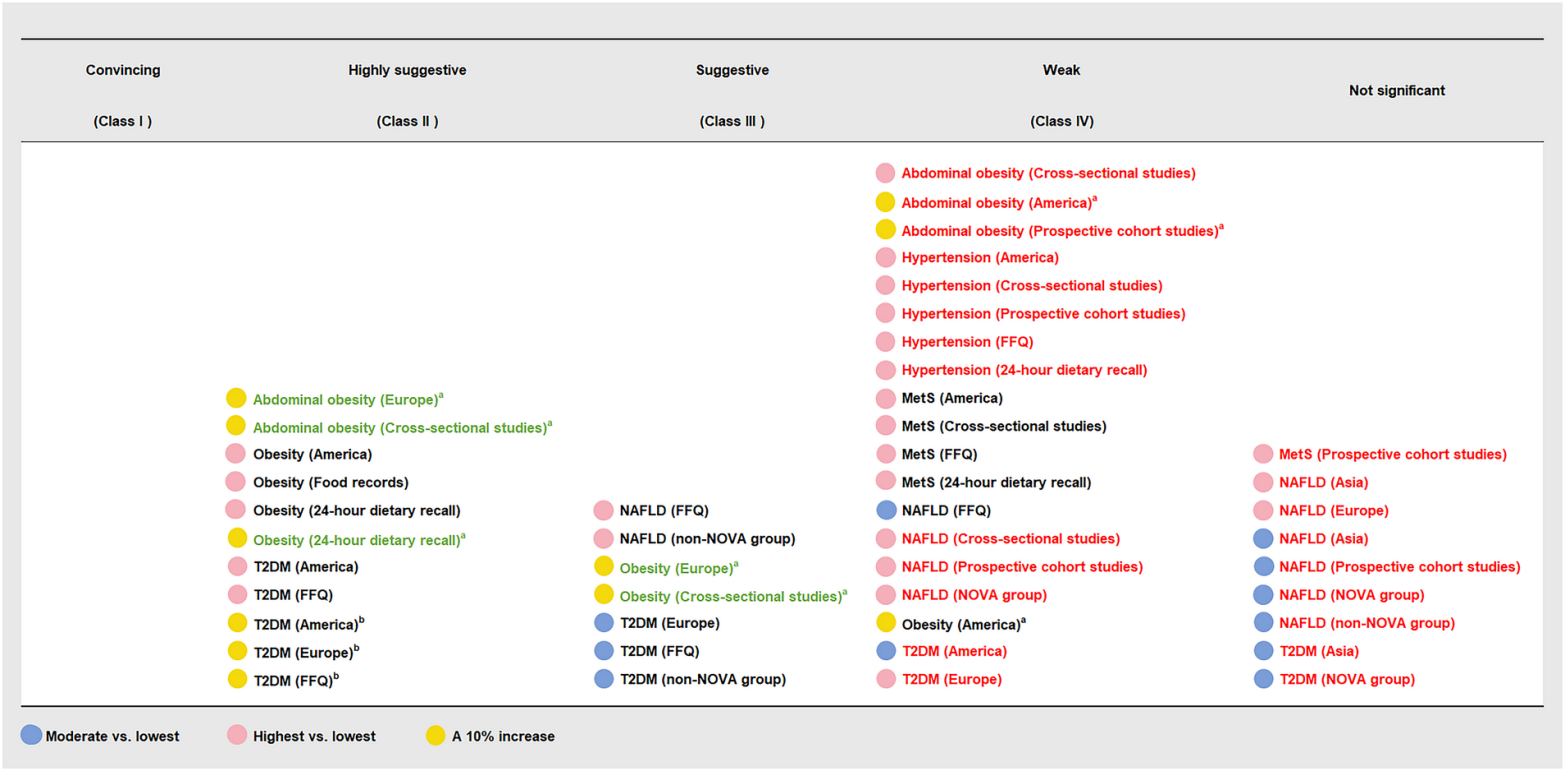
Subgroup analyses of credible assessment of meta-analyses evaluating the association between ultra-processed food consumption and metabolic disease risk. FFQ, food frequency questionnaire; MetS, metabolic syndrome; NAFLD, non-alcoholic fatty liver disease; T2DM, type 2 diabetes mellitus. “Lowest” was defined as the lowest UPF consumption quantile, “moderate” was defined as the first exposure quantile, and “highest” was defined as the highest UPF consumption quantile. The color of the text reflected the change of credibility compared with the main analyses: black, unchanged; red, degraded; green, upgraded. ^a^ The unit of UPF consumption was % kcal/d. ^b^ The unit of UPF consumption was % g/d.

The findings of subgroup analyses are displayed in Figures 4, 5 and Table 3. In subgroup analyses, the majority of subgroups exhibited consistent direction and significance with the main analysis. Regarding credibility, the credibility of the association between UPF consumption and obesity risk was upgraded or unchanged when compared with the main analysis. The credibility of the association between UPF consumption and T2DM risk was unchanged in the majority of highest vs. lowest and dose–response meta-analyses, while it was degraded in several moderate vs. lowest meta-analyses. In addition, the credibility of associations between UPF consumption and hypertension and NAFLD risk was degraded in the majority of subgroups. Notably, the credibility of the association between UPF consumption and MetS risk was still weak in the majority of subgroups, but it was degraded to not significant in prospective cohort studies.

**Table 3:** Subgroup analyses of credible assessment of meta-analyses evaluating the association between ultra-processed food consumption and metabolic disease risk.

Outcomes (Reference)/Author Year (Subgroups)	No. of studies	Comparison^a^	Random *p*-value	No. of cases	95% PI	*I*^2^ (95% CI) (%)	Tau^2^	Largest study effect (95% CI)	SSE/ESB	Evidence class^d^
**Abdominal obesity** (13)										
Moradi 2023 (Cross-sectional studies)	3	Highest vs. lowest	2.00 × 10^−3^	13,751	0.13–14.61	74 (11–92)	0.02	1.21 (1.10–1.46)	No/Yes	Weak
Moradi 2023 (America)	3	A 10% increase^b^	5.00 × 10^−3^	12,434	0.75–1.44	85 (55–95)	0.00	1.02 (1.01–1.03)	No/Yes	Weak
Moradi 2023 (Europe)	3	A 10% increase^b^	1.11 × 10^−7^	4,577	0.92–1.23	0 (0–90)	0.00	1.06 (1.03–1.08)	No/Yes	Highly suggestive
Moradi 2023 (Cross-sectional studies)	3	A 10% increase^b^	1.83 × 10^−11^	13,751	0.95–1.17	0 (0–90)	0.00	1.06 (1.04–1.08)	No/Yes	Highly suggestive
Moradi 2023 (Prospective cohort studies)	3	A 10% increase^b^	0.03	3,260	0.67–1.62	82 (46–94)	0.00	1.02 (1.01–1.03)	No/Yes	Weak
**Hypertension** (14)										
Wang 2022 (America)	6	Highest vs. lowest	7.00 × 10^−3^	11,093	0.80–1.84	63 (10–85)	0.02	1.19 (1.03–1.38)	No/Yes	Weak
Wang 2022 (Cross-sectional studies)	5	Highest vs. lowest	3.00 × 10^−3^	6,239	0.78–2.33	44 (0–80)	0.02	1.19 (1.03–1.38)	No/No	Weak
Wang 2022 (Prospective cohort studies)	4	Highest vs. lowest	0.02	7,136	0.72–1.89	55 (0–85)	0.01	1.21 (1.06–1.37)	No/Yes	Weak
Wang 2022 (FFQ)	6	Highest vs. lowest	5.00 × 10^−3^	7,716	0.85–1.70	51 (0–81)	0.01	1.21 (1.06–1.37)	No/Yes	Weak
Wang 2022 (24-h dietary recall)	3	Highest vs. lowest	0.04	5,659	0.09–17.72	62 (0–89)	0.03	1.19 (1.03–1.38)	No/No	Weak
**MetS** (15)										
Shu 2023 (America)	6	Highest vs. lowest	0.01	6,393	0.76–1.91	87 (73–93)	0.02	1.00 (0.99–1.01)	Yes/No	Weak
Shu 2023 (Cross-sectional studies)	6	Highest vs. lowest	2.00 × 10^−3^	4,112	0.73–2.95	69 (28–87)	0.05	1.20 (1.07–1.35)	No/No	Weak
Shu 2023 (Prospective cohort studies)	3	Highest vs. lowest	0.15	4,537	0.21–5.82	87 (61–95)	0.01	1.00 (0.99–1.01)	No/No	Not significant
Shu 2023 (FFQ)	5	Highest vs. lowest	0.01	3,383	0.65–2.72	88 (75–94)	0.04	1.00 (0.99–1.01)	Yes/No	Weak
Shu 2023 (24-h dietary recall)	4	Highest vs. lowest	1.00 × 10^−3^	5,266	0.86–1.64	26 (0–72)	0.00	1.20 (1.07–1.35)	No/Yes	Weak
**NAFLD** (16)										
Henney 2023 (Asia)	3	Moderate vs. lowest	0.41	4,091	0.54–2.01	2 (0–90)	0.00	1.03 (0.95–1.11)	No/No	Not significant
Henney 2023 (Prospective cohort studies)	3	Moderate vs. lowest	0.09	8,988	0.82–1.29	0 (0–90)	0.00	1.03 (1.00–1.07)	No/No	Not significant
Henney 2023 (FFQ)	6	Moderate vs. lowest	0.03	12,065	0.99–1.08	0 (0–75)	0.00	1.03 (0.99–1.07)	No/No	Weak
Henney 2023 (NOVA group)	3	Moderate vs. lowest	0.10	8,752	0.82–1.29	0 (0–90)	0.00	1.03 (0.99–1.07)	No/No	Not significant
Henney 2023 (non-NOVA group)	4	Moderate vs. lowest	0.09	3,615	0.89–1.32	0 (0–85)	0.00	1.08 (0.98–1.19)	No/No	Not significant
Henney 2023 (Asia)	3	Highest vs. lowest	0.07	4,091	0.00–900.52	89 (72–96)	0.18	1.11 (1.03–1.21)	No/No	Not significant
Henney 2023 (Europe)	4	Highest vs. lowest	0.05	5,610	0.34–5.44	93 (84–97)	0.08	1.05 (1.02–1.09)	No/No	Not significant
Henney 2023 (Cross-sectional studies)	3	Highest vs. lowest	5.22 × 10^−13^	676	0.71–3.53	0 (0–90)	0.00	1.71 (1.43–2.03)	No/Yes	Weak
Henney 2023 (Prospective cohort studies)	3	Highest vs. lowest	0.04	8,988	0.29–4.34	80 (37–94)	0.01	1.05 (1.02–1.09)	Yes/Yes	Weak
Henney 2023 (FFQ)	8	Highest vs. lowest	1.71 × 10^−5^	12,675	0.87–2.12	90 (82–94)	0.03	1.05 (1.02–1.09)	Yes/No	Suggestive
Henney 2023 (NOVA group)	4	Highest vs. lowest	0.02	9,057	0.59–2.57	90 (77–96)	0.02	1.05 (1.02–1.09)	No/No	Weak
Henney 2023 (non-NOVA group)	5	Highest vs. lowest	7.07 × 10^−5^	3,920	0.72–3.49	79 (49–91)	0.05	1.21 (1.10–1.33)	Yes/Yes	Suggestive
**Obesity** (13)										
Moradi 2023 (America)	5	Highest vs. lowest	2.02 × 10^−7^	18,918	0.90–2.55	65 (7–87)	0.02	1.53 (1.29–1.81)	No/Yes	Highly suggestive
Moradi 2023 (Food records)	3	Highest vs. lowest	4.28 × 10^−11^	5,769	0.41–8.97	16 (0–91)	0.00	2.17 (1.64–2.70)	No/Yes	Highly suggestive
Moradi 2023 (24-h dietary recall)	3	Highest vs. lowest	5.91 × 10^−9^	13,612	0.66–3.01	0 (0–90)	0.00	1.53 (1.29–1.81)	No/Yes	Highly suggestive
Moradi 2023 (America)	4	A 10% increase^b^	0.02	11,822	0.87–1.26	92 (81–96)	0.00	1.00 (0.99–1.01)	No/No	Weak
Moradi 2023 (Europe)	3	A 10% increase^b^	8.67 × 10^−6^	3,178	0.79–1.62	0 (0–90)	0.00	1.18 (1.08–1.28)	No/No	Suggestive
Moradi 2023 (Cross-sectional studies)	5	A 10% increase^b^	9.92 × 10^−6^	13,305	0.97–1.21	61 (0–85)	0.00	1.05 (1.03–1.07)	Yes/Yes	Suggestive
Moradi 2023 (24-h dietary recall)	3	A 10% increase^b^	3.58 × 10^−9^	10,253	0.94–1.18	0 (0–90)	0.00	1.05 (1.03–1.07)	No/Yes	Highly suggestive
**T2DM** (17, 18)										
Delpino 2022 (America)	4	Moderate vs. lowest	0.04	11,129	0.70–1.88	54 (0–85)	0.01	1.21 (1.12–1.31)	No/No	Weak
Delpino 2022 (Asia)	3	Moderate vs. lowest	0.34	3,818	0.20–5.80	56 (0–88)	0.01	1.25 (1.06–1.47)	No/No	Not significant
Delpino 2022 (Europe)	11	Moderate vs. lowest	2.14 × 10^−4^	19,977	1.04–1.18	0 (0–60)	0.00	1.08 (0.98–1.19)	No/No	Suggestive
Delpino 2022 (FFQ)	16	Moderate vs. lowest	8.60 × 10^−5^	33,798	0.97–1.29	31 (0–62)	0.00	1.21 (1.12–1.31)	No/No	Suggestive
Delpino 2022 (NOVA group)	3	Moderate vs. lowest	0.06	1,301	0.56–2.17	0 (0–90)	0.00	1.13 (1.01–1.27)	No/No	Not significant
Delpino 2022 (non-NOVA group)	15	Moderate vs. lowest	9.45 × 10^−5^	33,623	0.97–1.30	34 (0–65)	0.00	1.21 (1.12–1.31)	No/No	Suggestive
Chen 2023 (America)	3	Highest vs. lowest	3.93 × 10^−10^	19,503	0.35–6.15	82 (45–94)	0.01	1.36 (1.26–1.46)	No/Yes	Highly suggestive
Chen 2023 (Europe)	4	Highest vs. lowest	4.00 × 10^−3^	2,429	0.58–3.09	75 (30–91)	0.03	1.21 (1.04–1.20)	No/Yes	Weak
Chen 2023 (FFQ)	5	Highest vs. lowest	3.69 × 10^−16^	20,806	1.10–1.99	66 (10–87)	0.01	1.36 (1.26–1.46)	No/Yes	Highly suggestive
Chen 2023 (America)	3	A 10% increase^c^	8.58 × 10^−31^	19,503	0.92–1.35	54 (0–87)	0.00	1.13 (1.11–1.15)	No/Yes	Highly suggestive
Chen 2023 (Europe)	4	A 10% increase^c^	1.32 × 10^−9^	2,429	1.04–1.25	0 (0–85)	0.00	1.12 (1.04–1.20)	No/Yes	Highly suggestive
Chen 2023 (FFQ)	5	A 10% increase^c^	6.94 × 10^−39^	20,806	1.07–1.17	34 (0–75)	0.00	1.13 (1.11–1.15)	No/Yes	Highly suggestive

CI, confidence interval; ESB, excess significance bias; FFQ, food frequency questionnaire; MetS, metabolic syndrome; NAFLD, non-alcoholic fatty liver disease; PI, prediction interval; SSE, small-study effects; T2DM, type 2 diabetes mellitus.

a “Lowest” was defined as the lowest UPF consumption quantile, “moderate” was defined as the first exposure quantile, and “highest” was defined as the highest UPF consumption quantile.

b The unit of UPF consumption was % kcal/d.

c The unit of UPF consumption was % g/d.

d The color of the text reflected the change of credibility compared with the main analyses: black, unchanged; red, degraded; green, upgraded.

**Figure 6:**
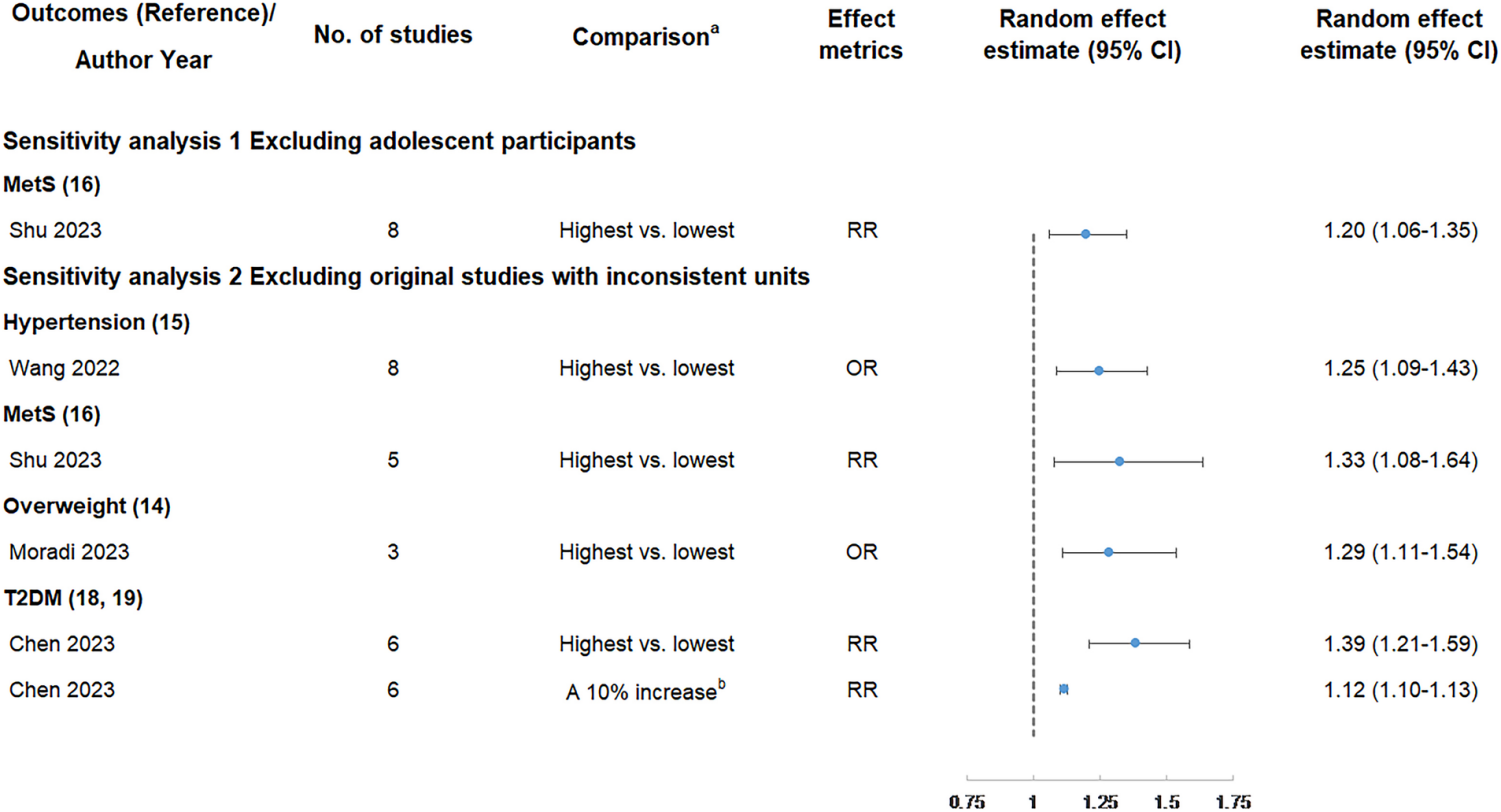
Sensitivity analyses of summary random effect estimate with 95% confidence interval from meta-analyses evaluating the association between ultra-processed food consumption and metabolic disease risk. CI, confidence interval; MetS, metabolic syndrome; NAFLD, non-alcoholic fatty liver disease; OR, odds ratio; RR, risk ratio; T2DM, type 2 diabetes mellitus. ^a^ “lowest” was defined as the lowest UPF consumption quantile, “moderate” was defined as the first exposure quantile, and “highest” was defined as the highest UPF consumption quantile. ^b^ The unit of UPF consumption was % g/d.

The results of sensitivity analyses are shown in Figure 6 and Table 4. In our study, only the association between UPF consumption and MetS risk included adolescents. After excluding adolescent participants, the positive association between UPF consumption and MetS risk was still classified as weak evidence. In addition, five meta-analyses could be further analyzed by excluding original studies with inconsistent units, and the results showed the direction and significance of these associations were all unchanged. However, the credibility of the association between UPF consumption and hypertension was degraded from suggestive evidence to weak evidence, and the credibility of the association between UPF consumption and T2DM (highest vs. lowest analysis) was degraded from highly suggestive evidence to suggestive evidence.

**Table 4:** Sensitivity analyses of credible assessment of meta-analyses evaluating the association between ultra-processed food consumption and metabolic disease risk.

Outcomes (Reference)/Author Year	No. of studies	Comparison^a^	Random *p*-value	No. of cases	95% PI	*I*^2^ (95% CI) (%)	Tau^2^	Largest study effect (95% CI)	SSE/ESB	Evidence class^c^
**Sensitivity analysis 1 Excluding adolescent participants**										
MetS (15)										
Shu 2023	8	Highest vs. lowest	4 × 10^−3^	8,635	0.83–1.73	83 (69–91)	0.02	1.00 (0.99–1.01)	Yes/No	Week
**Sensitivity analysis 2 Excluding original studies with inconsistent units**										
Hypertension (14)										
Wang 2022	8	Highest vs. lowest	1.00 × 10^−3^	11,673	0.86–1.80	58 (8–81)	0.02	1.19 (1.03–1.38)	No/Yes	Weak
MetS (15)										
Shu 2023	5	Highest vs. lowest	6.00 × 10^−3^	4,098	0.72–2.47	59 (0–85)	0.03	1.20 (1.07–1.35)	No/Yes	Week
Overweight (13)										
Moradi 2023	3	Highest vs. lowest	1.00 × 10^−3^	16,041	0.23–7.20	68 (0–91)	0.01	1.13 (1.08–1.41)	No/No	Week
T2DM (17, 18)										
Chen 2023	6	Highest vs. lowest	1.96 × 10^−6^	21,757	0.87–2.21	90 (81–95)	0.02	1.12 (1.04–1.20)	No/No	Suggestive
Chen 2023	6	A 10% increase^b^	5.25 × 10^−55^	21,757	1.09–1.15	16 (0–79)	0.00	1.13 (1.11–1.15)	No/Yes	Highly suggestive

CI, confidence interval; ESB, excess significance bias; MetS, metabolic syndrome; NAFLD, non-alcoholic fatty liver disease; PI, prediction interval; SSE, small-study effects; T2DM, type 2 diabetes mellitus.

a “Lowest” was defined as the lowest UPF consumption quantile, “moderate” was defined as the first exposure quantile, and “highest” was defined as the highest UPF consumption quantile.

b The unit of UPF consumption was % g/d.

c The color of the text reflected the change of credibility compared with the main analyses: black, unchanged; red, degraded; green, upgraded.

### Quality assessment of evidence and methods

The quality of 13 associations is shown in Supplementary Table S6. According to the GRADE, two (15.38%) associations were classified as moderate evidence, including the dose–response meta-analysis of overweight and obesity as well as T2DM. Four (30.77%) associations were classified as low evidence, including the dose–response meta-analysis of abdominal obesity and obesity, as well as the moderate vs. lowest meta-analysis of NAFLD and T2DM. Seven (53.85%) associations were classified as very low evidence, including the highest vs. lowest meta-analysis of abdominal obesity, hypertension, MetS, NAFLD, obesity, overweight, and T2DM.

The methodological quality of 6 systematic reviews is displayed in Supplementary Figure S1. According to the AMSTAR tool, four (66.67%) systematic reviews were categorized as high quality and the outcomes involved abdominal obesity, MetS, NAFLD, obesity, overweight, overweight and obesity, and T2DM (moderate vs. lowest meta-analysis). Two (33.33%) systematic reviews were categorized as moderate quality, and the outcomes involved hypertension and T2DM (highest vs. lowest and dose–response meta-analyses).

The two investigators showed a high consistency in quality assessment of evidence and methods, with a kappa value of 0.72 and 1.00, respectively.

## Discussion

In this UR, we performed a comprehensive overview of 13 meta-analyses to critically assess the credibility of associations between UPF consumption and metabolic disease risk. Overall, we observed significant positive associations in all 13 meta-analyses, with highly suggestive evidence supporting the association between UPF consumption and obesity and T2DM risk. Our findings demonstrated that UPF consumption might contribute to metabolic disease.

### Principal findings and possible explanations

In this study, the association between the highest UPF consumption and a 1.55-fold increased risk of obesity was supported by highly suggestive evidence. Although the above association was summarized from 7 cross-sectional studies, a similar trend was also observed in several prospective cohort studies (46, 47). For example, a prospective cohort study of 22,659 adults in the UK Biobank demonstrated that UPF consumption was associated with a 1.79-fold increased risk of obesity (46). In addition, another prospective cohort study of 17,310 adults in South Korea found that there was a significant positive association between UPF consumption and obesity risk (47). Meanwhile, a randomized controlled trial (RCT) study demonstrated that a diet with a large proportion of UPF could cause excess calorie intake and weight gain (48); in contrast, eliminating UPF from the diet could decrease energy intake and lead to weight loss (48). Therefore, UPF consumption may be a risk factor for obesity, and reducing UPF consumption may decrease the risk of obesity. However, of note, our study found evidence of ESB in the association between the highest UPF consumption and obesity risk, suggesting that the harmful association between UPF and obesity might have been exaggerated. Similarly, another dose–response meta-analysis included in our study suggested that UPF consumption was only associated with a 1.07-fold increased risk of obesity. Nevertheless, considering the high prevalence of obesity worldwide (49), a smaller effect size of UPF consumption may have important public health implications. Therefore, when formulating dietary guidelines in the future, it may be necessary to advocate reducing UPF consumption to decrease the incidence of obesity (19).

In addition, two meta-analyses based on prospective cohort studies were supported by highly suggestive evidence, indicating that UPF consumption was associated with T2DM in the highest vs. lowest and dose–response meta-analyses. In addition, in line with our findings, another meta-analysis excluded due to overlap also showed that there was a significant positive association between UPF consumption and T2DM risk (50). In addition, a UR evaluating the role of diet in T2DM demonstrated that several specific types of UPF, such as processed meat and sugar-sweetened beverages, could increase the incidence of T2DM (51). Therefore, the above evidence hints that UPF consumption may be a risk factor for T2DM. Of note, our study also suggests that dose may be an important factor influencing the effects of UPF consumption. In the moderate vs. lowest meta-analysis, the association between UPF consumption and T2DM risk was only supported by suggestive or weak evidence. In addition, the above-mentioned association was even not significant in the subgroups of the Asian population. This imply that the association between UPF consumption and T2DM risk may vary across different populations. Nevertheless, our findings should still be interpreted with caution. Although significant results were observed in the American and European populations and no significant findings were found in the Asian population, this discrepancy may be related to the level of UPF consumption being much lower among Asians than other populations. Meanwhile, only three studies were included in the association of the Asian population, with a relatively small sample size and short follow-up time. Furthermore, of note, the majority of original studies included in the moderate vs. lowest meta-analysis of the association between UPF consumption and T2DM risk did not define UPF based on the NOVA classification system. Further subgroup analyses showed that although the above-mentioned association was still significant in the non-NOVA group, the results were not significant in the NOVA group. Not defining UPF based on the NOVA classification system may affect the results due to potential misclassification bias, therefore more studies are needed to further explore the above-mentioned association.

Furthermore, we found that UPF consumption was associated with increased risks of hypertension, NAFLD, and MetS, and these associations were supported by suggestive or weak evidence. The credibility of associations between UPF consumption and hypertension and NAFLD did not seem to be robust as the grade of evidence was degraded in the majority of subgroups. Of note, the association between UPF consumption and NAFLD risk included more than half of the original studies that did not define UPF based on the NOVA classification system. However, the results of subgroup analyses showed that whether to define UPF based on the NOVA classification system may have an impact on the credibility of the association. The credibility of the association between UPF consumption and MetS risk was still classified as weak in the majority of subgroups, but became not significant when limited to prospective cohort studies only. Therefore, further studies are needed to explore the association between UPF consumption and MetS risk as prospective cohort studies are generally considered to provide higher levels of evidence than other observational studies (31).

The biological mechanisms of the association between UPF consumption and metabolic disease risk are still unclear, but there are several scenarios to explain the above-mentioned association. First, at the nutritional level, UPF usually has a poor nutritional profile and tends to be rich in added sugars, saturated fats, and sodium, as well as poor in fiber and micronutrients (52, 53). Evidence suggests that the poor nutritional profile can increase chronic and low-grade systemic inflammation, and then increase the risk of obesity and related metabolic disease such as T2DM and NAFLD (54). In addition, at the food level, UPF consumption can replace unprocessed or minimally processed food consumption (55). According to the NOVA classification system, unprocessed or minimally processed food is fresh or processed by industrial processes such as removal of unwanted or inedible parts, boiling, drying, roasting, freezing, and refrigeration (10). None of these processes add salt, sugar, oils or fats, or other food substances to the original food, and examples include fresh vegetables and fruits, grains, legumes, pasteurized milk, yogurt without added sugar, nuts and seeds without added sugar, etc. (10). Of note, unprocessed or minimally processed food may exert a protective effect against metabolic disease (56–58). On the one hand, this may be associated with health-promoting components. For example, fresh vegetables and fruits provide antioxidants which may help to prevent inflammation and oxidative stress in metabolic disease (59). On the other hand, beyond single foods or nutrients, the overall dietary pattern may also explain the association between diet and health (60). For instance, Greater adherence to the prudent pattern rich in vegetables and fruits, whole grains, and legumes can predict lower risks of MetS (61).

The aforementioned evidence suggests that nutritional factors can explain the association between UPF consumption and metabolic disease risk. Additionally, food processing factors may also play an important role during this process (57). At the food processing level, the loss of physical and structural characteristics of the food matrix is linked with a lower satiety potential (62), combined with the non-nutritional features of UPF such as delicious and ready-to-eat, UPF may lead to continuous and unconscious eating behaviors and then further increase the risk of metabolic disease (13, 63). In addition, food additives may create an environment in the gut that favors the selection of microbes that promote inflammation-related disease (64). Furthermore, neo-formed compounds resulting from food processing (e.g., advanced glycation end products) are associated with an increased risk of metabolic disease (65). Similarly, chemical contaminants released from food packaging (e.g., phthalates and bisphenol A) are known as endocrine-disrupting chemicals and are involved in the pathophysiological processes of various diseases (66).

### Strengths and limitations

To our knowledge, this is the first UR to provide a comprehensive summary of the associations between UPF consumption and metabolic disease from published systematic reviews with meta-analyses and to further analyze the robustness of these associations through a series of subgroup and sensitivity analyses. According to standard criteria, we comprehensively assessed the credibility and quality of each meta-analysis. Our findings demonstrated that UPF consumption might be a risk factor for metabolic disease, providing a basis for clinicians and public health policymakers to make decisions. In addition, our study also found the shortcomings of the current research and provided study directions for future analyses.

Nevertheless, our UR also had several limitations. First, as the UR is a method used to summarize the evidence from meta-analyses, the reliability of the UR relies heavily on the included meta-analyses and their original studies. Potential issues in meta-analysis and original studies may affect the analysis of UR. Second, as the UR only evaluates published meta-analyses with available data, meta-analyses that lack specific data or include less than three original studies as well as individual studies that have not been summarized by meta-analyses may be ignored. Therefore, future meta-analyses should report data in detail and focus on outcomes that had not yet been evaluated by our UR, such as dyslipidemia and hyperuricemia (56, 67, 68). In addition, due to the lack of RCTs, our UR only included meta-analyses of observational studies. Therefore, residual confounding and measurement errors were unavoidable. Meanwhile, due to the nature of observational studies and factors for quality downgrade, the quality of most associations was only classified as low or very low based on the GRADE. Third, the most commonly used dietary assessment method in the included original studies was FFQ, however, there was no study specifically designed FFQ based on the NOVA classification system. In addition, several meta-analyses simultaneously included original studies that defined UPF based on the NOVA classification system and original studies that did not define UPF based on the NOVA classification system (16, 17). These might lead to the misclassification of UPF, thereby leading to biased associations. Furthermore, specific types of UPF may have different effects on the results, but this was not been considered in our study. Fourth, the majority of included original studies divided UPF consumption into tertiles, quartiles, or quintiles, rather than pre-defined cut-off values. These inconsistencies in classification might limit conclusions about how much UPF consumption was needed in the diet to trigger adverse metabolic disease. In addition, the units used to assess UPF consumption were not consistent in some meta-analyses, therefore this might be an important source of heterogeneity and limit the interpretation of the results as well as the comparability of different meta-analyses. Meanwhile, our sensitivity analyses also suggested that the unit of UPF might have an impact on the credibility of the associations. In this case, it is important for original studies to unify the units of UPF. According to our UR, an energy ratio was widely used, but recent studies suggested that a weight ratio might be more appropriate than an energy ratio to assess UPF consumption as it accounted for UPF that did not provide energy (69, 70). Nevertheless, there was a view that the weight ratio was also flawed, and no ideal weighting method exists at present (71). Hence, this issue needs to be explored in future studies. Furthermore, it is very important for original studies to reasonably determine the confounding factors that need to be adjusted. For example, body mass index which might be a potential mediator variable had been adjusted in most of the original studies. In this case, the effect size represented the effect after deducting the influence of body mass index rather than the overall effect. Therefore, we suggest that the causal directed acyclic graphs can be used in future studies to more reasonably determine the confounding factors that need to be adjusted. Last, considering that the existing studies were mainly conducted in Brazil, the United States and several European countries, the extrapolation of the results might be limited.

## Conclusion

In conclusion, highly suggestive evidence indicated that UPF consumption was associated with increased risks of obesity and T2DM. The associations between UPF consumption and other metabolic disease need to be further explored. In addition, considering that there are still many limitations in the existing original studies and meta-analyses. Therefore, more well-designed original studies and meta-analysis are needed to verify our findings in the future.

## Data availability statement

The original contributions presented in the study are included in the article/Supplementary material, further inquiries can be directed to the corresponding authors.

## Author contributions

J-LL: Writing – original draft. Y-FW: Writing – original draft. J-NS: Writing – original draft, Data curation. Y-CS: Writing – original draft, Data curation. F-HL: Writing – original draft, Data curation. M-HS: Writing – original draft, Data curation. QC: Writing – review & editing. Q-JW: Writing – review & editing. Y-HZ: Writing – review & editing.
