# Supplementary figures and images for: Ultra-processed food consumption and metabolic disease risk: an umbrella review of systematic reviews with meta-analyses of observational studies
Source: Front Nutr. 2024 Jan 31;11:1306310. doi: 10.3389/fnut.2024.1306310 (PMC10864658; doi:10.3389/fnut.2024.1306310)

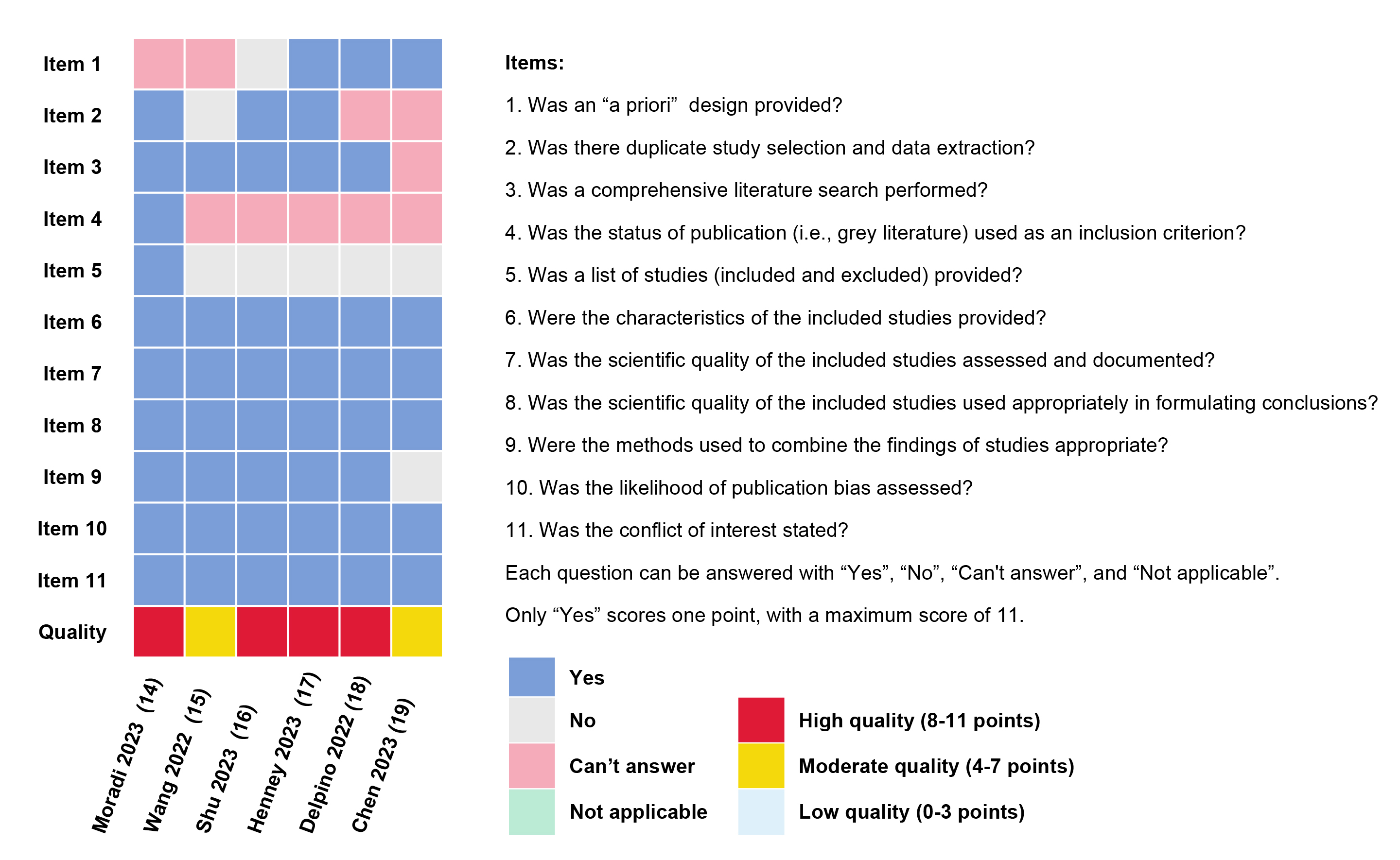

Supplement: Supplementary file 2 [file Image_1.JPEG]
